# Deaths

**Published:** 1915-08

**Authors:** 


### Deaths.

The many friends of Dr. M. M. Carrick will be sorry to learn of the death of his mother, Mrs. Cammie R. Howard, which occurred in Dallas, at St. Paul's Sanitarium, Thursday evening, July 8th. Mrs. Howard moved to Dallas in 1889 and has since made that city her home. She lost two sons, who contracted tuberculosis, and since that time Mrs. Howard had devoted considerable time and money towards aiding the tubercular worthy poor. It was through her influence that a State hospital for tubercular victims was established at Carlsbad, Texas, she appearing before the Legislature in behalf of this cause. She also took much interest in the establishment of a Dallas city and county tubercular hospital. Mrs. Howard was a graduate of the Methodist Female College at Mansfield, Louisiana, and had an A. B. degree from the Newcomb College, the women's college of Tulane University, New Orleans. She was a member of St. Matthew's cathedral and took much interest in the charity work of this church.

